# Retraction: APRIL Induces Tumorigenesis and Metastasis of Colorectal Cancer Cells via Activation of the PI3K/Akt Pathway

**DOI:** 10.1371/journal.pone.0222525

**Published:** 2019-09-12

**Authors:** The PLOS ONE Editors

After publication of this article [1], concerns were raised about the following results:

- In Figure 2E, several cells and groups of cells appear to be repeated within and across panels.
- In Figure 3F, the shAPRIL/MMP-2 panel appears similar to the shAPRIL/CDK4 panel, and the lower left corner of these two panels appears similar to the upper right corner of the shAPRIL/MMP-9 panel.
- The shAPRIL/Ki-67 and shNTC/Ki-67 panels of Figure 3F in [1] appear similar to ki67 panels B and C, respectively, in Figure 2C of [2] and to the PCNA H-siAPRIL and Control panels, respectively, in Figure 8b of [3].
- The tumor volumes reported in Figure 3 are high relative to limits commonly applied in mouse tumor studies.

In addition, there is text in the Introduction and Discussion sections of [1] that overlaps with previously published work, including [4–6].

The corresponding author confirmed the concerns about Figures 2E and 3F, and noted that the experiments reported in Figure 2D, E were conducted by a third-party company whose contributions were not disclosed in the published article [1]. The original image data to support Figures 2E, 3F, and other results reported in the article are no longer available. The authors replicated the experiments in question but in the absence of the original data, we have been unable to clarify the concerns about the published figures.

In light of the above concerns, the *PLOS ONE* Editors retract the article.

The corresponding author notified the journal that all authors agree to the retraction. The other authors either could not be reached or did not reply directly.

Additionally, at the time of the article’s [1] publication, permissions were not obtained to use and offer the images in the shAPRIL/Ki-67 and shNTC/Ki-67 panels of Figure 3F under the CC-BY license. *PLOS ONE* has obtained permissions from Springer Nature to retain these images in the retracted article under a non-CC-BY license. The images in the shAPRIL/Ki-67 and shNTC/Ki-67 panels of Figure 3F [1] are excluded from the article’s CC-BY license. The article [1] was republished at the time of retraction to update the Copyright Statement and add information about the original source and reproduction permissions for these images to the Figure 3 legend; the full reference for the Wang et al. (2012) article [2] has also been added to the References list in [1] as item 35. Please see the complete, correct Figure 3 caption here:

**Figure 3**. **APRIL knockdown increases tumor growth and promotes metastasis in vivo**.

(A) Nude mice were subcutaneously injected in the right flank with control cells, shNTC transfected cells and shAPRIL (sh637) transfected cells. (B) A sample tumor from each group is shown. (C) Tendency of tumor growth after injection in nude mice in different groups. Tumor volume was measured by vernier caliper in cm3. (D) Total numbers of metastatic liver nodules (>0.5 mm) in individual mice were counted under a surgical telescope. Representative hematoxylin and eosin staining of 4-μm sections of livers from shAPRIL and shNTC groups are shown. Arrow indicates tumor nodules. Scale bar, 100 μm. (E) Western blot and immunohistochemistry of APRIL in tumors of nude mice from each group. (F) Immunohistochemical analysis of p-Akt, p-mTOR, Ki-67, cyclin D1, CDK4, p-Rb, MMP-2 and MMP-9 in tumors from nude mice injected with shNTC or shAPRIL SW480 cells. Scale bar, 40 μm. The shAPRIL/Ki-67 and shNTC/Ki-67 panels of Figure 3F report *Material from*: Wang et al., Targeting of colorectal cancer growth, metastasis, and anti-apoptosis in BALB/c nude mice via APRIL siRNA, published 2012 [Springer Science+Business Media, LLC. 2011][35] reproduced with permission of SNCSC. The images in the shAPRIL/Ki-67 and shNTC/Ki-67 panels of Figure 3F are excluded from this PLOS ONE article’s CC-BY license.
